# 

**Published:** 1903-12-05

**Authors:** 


### CONTENTS.

**Mortality of Infectious Diseases** - 161
**Pyrexia in Cancer of the Liver** - 162
**Annotations** - 163
**Notes and News** - 164
**Hospital Clinics and Medical Progress—**
What We Owe to Experiments on Animals - 165
The Spread of Plague by Fleas - 166
Injuries of Nerves - 167
Gonococcus Infection in Infants - 167
PROGRESS IN SURGERY - 168
PROGRESS IN DISEASE OF DIGESTIVE ORGANS - 169
PROGRESS IN ANÆSTHETICS - 170
**The Treatment of the Sick in Norwich during the Seventeenth Century** - 172
**The Royal Society** - 172
**Hospital Administration—**
Modern Hospital and Institutional Fittings.—III - 173
Construction Notes - 174
St. Bartholomew's Hospital - 175
Belgrave Hospital for Children - 178
The Salop Infirmary - 178
Practical Departments - 179
**Scraps and Gleanings** - 180
**The Student of Medicine** - 180

*NURSING SECTION.*

**Notes on News from the Nursing World—** Our Christmas Distribution—The Junius S. Morgan Benevolent Fund—Queen Alexandra's Military Nursing Service—Resignations and Dismissals at Charing Cross Hospital—Appointments to Camberwell Infirmary—The Bishop of Stepney on Nursing—The Perils of Starting a Nursing Home—A Change of Title—The Central Midwives Board and its Certificate—Monument to Spanish-American War Nurses—Alleged "Menacing Letters"—A Piano for Workhouse Nurses—Retrogression at Loughton—Queen's Nurses at Loughborough—Nursing Homes and the Companies Act—What's in a Name—Short Items - 123-126

**The Nursing Outlook** - 127

**Lectures on Ophthalmic Nursing** - 128

**History of a Nursing Home** - 129

**The Nurses of St. George's in the East** - 130

**Anglo-American Nursing Home, Rome** - 131

**Where to Go** - 132

**A Training School for Nurses in Naples** - 132

**The Night Superintendent** - 133

**Appointments** - 133

**Echoes from the Outside World** - 134

**Notes and Queries** - 135

**Everybody's Opinion** - 133

**For Reading to the Sick** - 136

